# Effect of capsule invasion on recurrence and survival in axillary lymph node metastases of breast cancer

**DOI:** 10.1590/1806-9282.20251372

**Published:** 2026-05-01

**Authors:** Nurhilal Kızıltoprak, Ayşe Gökçen Sade, Bülent Güleç

**Affiliations:** 1 Sultan Abdulhamid Han Educational and Research Hospital – Istanbul, Türkiye.

**Keywords:** Breast cancer, Breast neoplasms, Lymphatic metastasis, Lymph nodes, Neoplasm recurrence, local

## Abstract

**OBJECTIVE:**

The aim of this study was to investigate the effect of capsule invasion on recurrence and survival in breast cancer patients with axillary lymph node metastases.

**METHODS:**

This retrospective study included 135 breast cancer patients with axillary lymph node involvement who underwent surgery between 2009 and 2018. The relationships between capsule invasion and various clinicopathological factors—including demographic parameters, tumor stage, surgical technique, histological type, number of involved lymph nodes, tumor size, estrogen receptor, progesterone receptor, human epidermal growth factor receptor 2, and Ki-67 index—were analyzed using chi-square, Kaplan-Meier, and Fisher's exact tests. Additionally, multivariate Cox regression analysis was performed to assess the independent prognostic value of capsule invasion for recurrence. A p<0.05 was considered statistically significant.

**RESULTS:**

Capsular invasion was observed in 64 of 135 patients (47.4%). Recurrence occurred in six patients with capsule invasion compared to only one patient without capsule invasion. Multivariate Cox regression analysis, controlling for positive lymph node count, tumor size, grade, and receptor status, confirmed that capsule invasion was an independent predictor of recurrence [HR 3.45, 95%CI 1.12–10.65, p=0.032]. No significant association was found between tumor size and capsule invasion (p>0.05). During follow-up, 20 patients died (9 with and 11 without capsule invasion), with no significant difference in 5-year survival or mean survival time between groups (p=0.972, Kaplan-Meier analysis). Grading of capsular invasion showed a significant correlation with recurrence (p=0.026).

**DISCUSSION:**

Although lymph node capsule invasion in breast cancer with axillary lymph node involvement does not significantly impact overall survival, it independently and significantly elevates the risk of recurrence, as demonstrated by multivariate analysis.

## INTRODUCTION

Axillary lymph node metastasis is a major prognostic factor in breast cancer, with the number of involved nodes strongly associated with recurrence and survival. Accordingly, meticulous evaluation of the axillary region is crucial. Another relevant factor is capsule invasion of metastatic lymph nodes, which significantly influences recurrence rates. Some studies also suggest a relationship between capsule invasion and increased axillary tumor burden^1^, including analyses of extranodal extension (ENE) size and its association with clinical parameters.

Although several studies have examined ENE in breast cancer, the existing evidence regarding its prognostic value remains inconsistent, particularly in relation to recurrence and overall survival.

Moreover, the extent of capsule invasion has not been standardized, and—unlike in oral squamous cell carcinoma—it is not incorporated into the American Joint Committee on Cancer (AJCC) staging system for breast cancer, leaving its clinical relevance uncertain.

These gaps highlight the need for additional studies aimed at clarifying the prognostic impact of capsule invasion in axillary lymph node—positive breast cancer.

In this retrospective study of 135 patients, we aimed to investigate the impact of capsule invasion on recurrence and survival, while also exploring its association with other clinical and pathological features. Previous studies have demonstrated a link between ENE and both mortality and recurrence^2,3^, suggesting that capsule invasion may merit inclusion in oncological staging systems. Notably, the 8th edition of the AJCC incorporates capsule invasion into N staging for oral squamous cell carcinoma but not for breast cancer^4^.

Capsule invasion has also been linked to increased lymph node metastatic burden^5,6^. Specifically, ENE in a positive sentinel lymph node (SLN) may reflect additional tumor burden beyond the SLN, though its prognostic significance remains uncertain.

## METHODS

This retrospective study was approved by the Clinical Research Ethics Committee of the University of Health Sciences (Decision No: 41017, Date: 17.06.2021). A total of 135 patients who underwent breast cancer surgery between February 2009 and October 2018 with confirmed axillary lymph node metastasis were included. Exclusion criteria comprised patients without carcinoma, without axillary involvement, or with inaccessible medical records.

All patients received adjuvant oncologic treatment according to National Comprehensive Cancer Network (NCCN) guideline recommendations and institutional protocols, including systemic chemotherapy, radiotherapy, and/or endocrine therapy as indicated. Owing to the retrospective design and the decade-long study period (2009–2018), detailed data on specific adjuvant regimens and treatment timing were not uniformly available, reflecting evolving oncologic practice standards during this time.

Data were obtained retrospectively from hospital records. Patient follow-up information was collected through outpatient evaluations and oncology clinic records, enabling assessment of recurrence and survival outcomes.

### Clinicopathological variables

Evaluated parameters included patient age, tumor size, number of metastatic axillary lymph nodes, histological type, tumor grade, capsular invasion status, estrogen receptor (ER) and progesterone receptor (PR) positivity, and human epidermal growth factor receptor 2 (HER2) status. ER status was defined using the Allred scoring system, with scores ≥3 considered positive. HER2 positivity was determined by immunohistochemistry 3+ staining or HER2 gene amplification confirmed by fluorescence in situ hybridization.

### Statistical analysis

Statistical analyses were performed using Statistical Package for the Social Sciences version 25.0 (IBM Corp., Armonk, NY, USA). Normality of continuous variables was assessed using Kolmogorov-Smirnov and Shapiro-Wilk tests. Group comparisons were conducted using Fisher's exact test, chi-square test, and Student's t-test, as appropriate. To evaluate the independent prognostic value of capsule invasion for recurrence, multivariate analysis was performed using Cox proportional hazards regression. The model included capsule invasion, number of positive lymph nodes, tumor size, tumor grade, and hormone receptor status (ER/PR). Results are presented as hazard ratios (HRs) with 95%CIs. A p<0.05 was considered statistically significant. This study was conducted in accordance with the principles of the Declaration of Helsinki.

## RESULTS

A total of 329 patients underwent breast cancer surgery in our center between February 2009 and December 2018. Among these patients, 135 exhibited metastases in the axillary lymph nodes, while 194 showed no evidence of axillary metastasis.

Demographic characteristics of the patients, Tumor, Node, Metastasis classification staging, laterality and quadrant of the tumor, multifocality, surgical method, tumor histology, capsule invasion, recurrence, and survival information are shown in Table 1.

**Table 1:** Baseline characteristics of the study group (n=135).

Parameter		n (%)
Age		
	Minimum	28
	Maximum	84
	Mean±SD	56.98±14.33
Gender		
	Female	132 (97.7%)
	Male	3 (2.3%)
pT stage		
	T1	37 (27.4%)
	T2	83 (61.5%)
	T3	11 (8.1%)
	T4	4 (3.0%)
pN stage		
	N1	74 (54.8%)
	N2	36 (26.7%)
	N3	25 (18.5%)
Metastasis (M)		
	M0	98 (72.6%)
	M1	37 (27.4%)
Stage		
	Stage 2	57 (42.2%)
	Stage 3	41 (30.4%)
	Stage 4	37 (27.4%)
Laterality		
	Right breast	68 (50.4%)
	Left breast	67 (49.6%)
Tumor location (quadrant)		
	Upper outer	79 (58.5%)
	Upper inner	12 (8.9%)
	Lower outer	13 (9.6%)
	Lower inner	11 (8.1%)
	Retroareolar	16 (11.9%)
	Multifocal	4 (3.0%)
Type of surgery		
	Breast-conserving surgery (BCS)	15 (11.1%)
	Mastectomy	120 (88.9%)
Tumor histology		
	Invasive ductal carcinoma	102 (75.6%)
	Invasive lobular carcinoma	23 (17.0%)
	Papillary carcinoma	1 (0.7%)
	Medullary carcinoma	6 (4.4%)
	Apocrine carcinoma	3 (2.2%)
Capsule invasion		
	Present	64 (47.4%)
	Absent	71 (52.6%)
Recurrence		
	Present	7 (5.2%)
	Absent	128 (94.8%)
Survival status at last follow-up		
	Alive without disease (no evidence of disease)	93 (68.9%)
	Alive with disease (persistent/recurrent disease)	22 (16.3%)
	Dead (all causes)	20 (14.8%)

SD: standard deviation; BCS: breast-conserving surgery.

An examination of the pathological T stages in 135 patients revealed that 37 were classified as T1, 83 as T2, 11 as T3, and 4 as T4. It has been observed that lymph node metastasis can be seen even in early-stage breast tumors.

Among the patients, three were male, and 132 were female. Eleven of the female patients were in the pre-menopausal period.

Breast-conserving surgery was performed in 15 patients, and mastectomy was performed in 120 patients. Thirteen patients underwent only SLN biopsy, while 122 patients underwent axillary dissection.

Based on the presence of capsule invasion, gender, age, laterality, estrogen positivity, progesterone positivity, cerbB2 positivity, stage, tumor histology, tumor size, focality, mean survival, exitus status, and triple negativity status are shown in Table 2.

**Table 2:** Comparative analysis of clinicopathological features and recurrence patterns according to the presence and grade of capsule invasion.

Parameter	Capsule invasion present (n=64)	No capsule invasion (n=71)	p
Gender			
Female	62	70	0.6
Male	2	1	
Age (mean±SD)	57.73±14.6	56.3±14.1	0.56
Breast cancer laterality			0.96
Right	32	36	
Left	32	35	
ER positivity			0.957
Negative	15	16	
Positive	49	55	
PR positivity			0.779
Negative	25	26	
Positive	34	39	
cerbB2 positivity			0.958
Negative	40	41	
Positive	22	23	
Ki-67 (average)	27.52%	26.59%	0.906
Pathological stage			0.108
Stage 2	21	36	
Stage 3	23	18	
Stage 4	20	17	
Tumor histology			0.73
Invasive ductal carcinoma	51	51	
Invasive lobular carcinoma	10	13	
Papillary carcinoma	0	1	
Medullary carcinoma	2	4	
Apocrine carcinoma	1	2	
Tumor diameter (mm)	29.89	32.3	0.415
Focality			0.461
Unifocal	53	62	
Multifocal	11	9	
Average survival (years)	12.547	12.332	0.972
Exitus/alive			
Exitus	9	11	
Alive	55	60	
Triple negativity			0.733
Triple negative	8	7	
Not triple negative	54	64	
Number of malignant LNs excised	6.91±6.11	3.82±4.33	0.01
Presence of recurrence			0.032
No recurrence	58	70	
Recurrence detected	6	1	
Capsule invasion grade			
	No recurrence	Recurrence detected	Total
0	8	0	8
1	6	2	8
2	10	1	11
3	8	0	8
4	1	2	3

Statistical analysis of capsule invasion grade and recurrence was performed using Fisher's exact test (p=0.026). SD: standard deviation; LN: lymph node; ER: estrogen receptor; PR: progesterone receptor.

Among the 20 patients who died, nine were in the capsular invasion group. The mean survival time was approximately 12 years in both groups. Kaplan-Meier analysis using Log-Rank, Breslow, and Tarone-Ware tests did not detect a statistically significant difference in overall survival between patients with and without capsule invasion (p=0.972). However, this finding should be interpreted with caution, as the study is underpowered to detect survival differences due to the limited number of death events (n=20). Five-year survival rates appeared similar between groups. The Kaplan-Meier survival curves were revised to include 95%CIs, providing a clearer representation of survival variability between groups.

Recurrence occurred in seven patients (5.2%) overall. The recurrence rate was significantly higher in patients with capsular invasion (6/64, 9.4%) compared to those without invasion (1/71, 1.4%; p=0.043, Fisher's exact test). Multivariate Cox regression analysis, controlling for positive lymph node count, tumor size, grade, and hormone receptor status, confirmed that capsular invasion was an independent predictor of recurrence (HR 3.45, 95%CI 1.12–10.65, p=0.032). A systematic distribution of recurrence sites according to capsule invasion status is presented in Table 3.

**Table 3:** Pattern of recurrence according to capsule invasion status.

Recurrence site	Capsule invasion (+) (n=6)	Capsule invasion (-) (n=1)	Total
Local	1	0	1
Regional	3	0	3
Distant	2	1	3
Total	6	1	7

The clinical characteristics of the seven patients with recurrence are summarized below:

1. A 52-year-old female (Stage IV, T2N1M1, HER2-positive) developed supraclavicular recurrence after receiving axillary clearance (AC), Taxol, and radiotherapy.
2. A 51-year-old female (Stage IV, T3N1M1, Luminal A) had axillary recurrence; no radiotherapy was administered.
3. A 73-year-old female (T1N1M0, HER2-positive) developed axillary recurrence after receiving monoclonal antibody, chemotherapy, and radiotherapy.
4. A 51-year-old female (Stage II, T1N1M0, HER2-positive) experienced ipsilateral breast recurrence following breast-conserving surgery and full adjuvant treatment.
5. A 74-year-old male (Stage IV, T2N2M1, Luminal A) had recurrence despite receiving chemotherapy, hormonal therapy, and radiotherapy.
6. A 78-year-old female (Stage IV, triple-negative) developed recurrence after systemic treatment excluding radiotherapy.
7. A 60-year-old female (Stage IV, HER2-positive) had recurrence and died 2 years later despite receiving AC, Taxol, Herceptin, and radiotherapy.

In the literature, the amount of extracapsular invasion is divided into five grades^7^:

- Grade 0: tumor within the nodal capsular sinus without thickening of the lymph node capsule
- Grade 1: tumor involving the subcapsular sinus with thickening of the lymph node capsule
- Grade 2: tumor extends ≤1 mm beyond the lymph node capsule
- Grade 3: tumor extends >1 mm beyond the lymph node capsule
- Grade 4: no more lymphatic tissue

**Figure 1:**
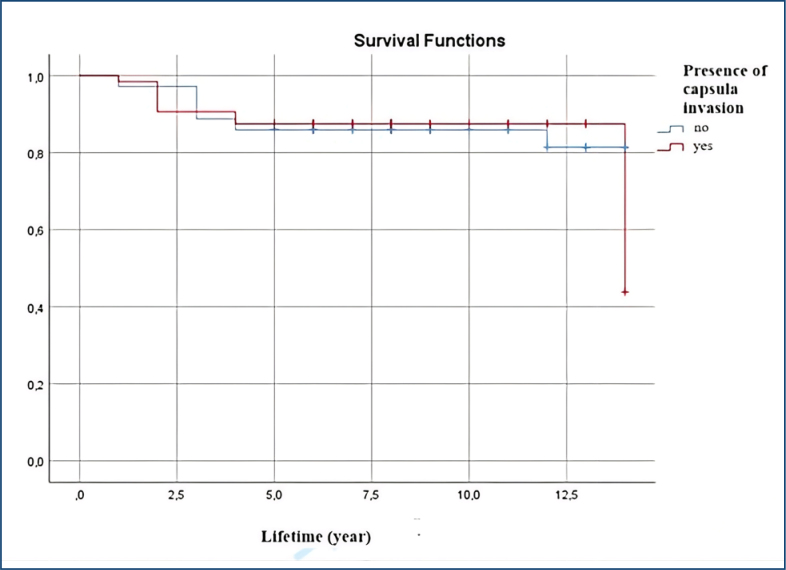
Kaplan-Meier survival curves with 95% confidence intervals according to the presence of capsule invasion.

Capsule invasion was graded in 38 patients, and a significant correlation with recurrence was found (p=0.026; Table 2). However, capsule invasion had no impact on survival (p=0.97; Figure 1).

## DISCUSSION

Invasive breast cancer is the most common malignancy in women, with lymph node metastasis being a major prognostic factor. While prognosis is known to correlate with the number of metastatic nodes, the prognostic impact of capsular invasion remains unclear. Fisher et al. first reported extranodal extension in 1976, suggesting its significance in axillary metastases^8^. Subsequent studies have linked the number of extranodal extension-positive nodes with prognosis^9–11^. Kustić and Yang et al. noted that extranodal extension predicts the number of metastatic nodes^9,10^, while Ahmad et al. associated perinodal spread with nodal burden^12^. Nottegar et al. found that extranodal spread increases mortality and recurrence risk^13^. Kanyılmaz et al. identified extracapsular spread as a prognostic factor in pT1–2 N1 breast cancer^7^. The present study demonstrates that lymph node capsular invasion is an independent predictor of recurrence in breast cancer patients with axillary involvement. Multivariate Cox regression analysis, controlling for nodal burden, tumor characteristics, and receptor status, confirmed that capsular invasion significantly increases recurrence risk (HR 3.45, 95%CI 1.12–10.65, p=0.032).

Yang et al. further classified patients into minimal extranodal extension group and extensive (EEE) extranodal extension group, showing higher nodal involvement in the EEE group (p<0.001), though prognostic value was not assessed^10^. Katz et al. proposed a grading system where extension >2 mm significantly increased local and regional recurrence risk compared to ≤2 mm^14^.

Extracapsular invasion is a key factor increasing the risk of metastasis in non-sentinel lymph nodes, prompting further research on its predictive value in such patients. Nowikiewicz et al. reported significant differences in pN stage and the number of metastatic nodes between patients with and without extracapsular extension^15^.

Ma et al.^4^ identified axillary extracapsular invasion as a prognostic factor associated with distant recurrence-free and overall survival. Although its presence correlated with high nodal burden, using 2 or 3 mm cut-offs to quantify extension did not yield prognostic significance.

Leonard et al.^16^ showed that extranodal spread reduced survival: 5- and 10-year survival rates were 70 and 39% with spread, versus 51 and 19% without. It was also an independent negative prognostic factor in patients with 1–3 involved lymph nodes.

Some studies^16^ have reported that extracapsular extension worsens survival, particularly in patients with limited nodal involvement, as its impact may be masked when total nodal burden is high.

Nowikiewicz et al.^15^ reported that extracapsular invasion was associated with a higher axillary metastatic burden and differing long-term outcomes.

Gooch et al.^17^ found extracapsular extension to be more frequent in patients with multifocal tumors, lymphovascular invasion, steroid hormone receptor positivity, and larger tumor size.

Schwentner et al.^18^ observed that extracapsular invasion adversely affected 5-year overall survival but not disease-free survival.

Kustić^9^ reported a significant association between extranodal spread and distant recurrence (p=0.013). Some studies suggest that spread <2 mm does not impact recurrence rates, aligning with findings in patients without spread^19,20^. However, these results should be interpreted cautiously due to retrospective design, limited axillary treatment data, and small sample sizes.

Extranodal spread is found in a significant proportion of both metastatic SLNs and non-SLNs, and is a negative prognostic factor for overall and recurrence-free survival^18^. Although there is no consensus on optimal treatment for patients with capsule invasion, further prospective studies are warranted due to its demonstrated prognostic impact.

The retrospective design spanning 2009–2018 may have introduced treatment heterogeneity, as adjuvant systemic therapy and radiotherapy protocols evolved during this decade (e.g., adoption of ACOSOG Z0011 criteria). Although all patients were treated in accordance with NCCN guideline recommendations, variations in specific adjuvant regimens may represent a potential confounding factor influencing recurrence and survival outcomes.

Additionally, the grading of capsule invasion could not be performed for all patients with documented invasion. Although capsule invasion was recorded as a binary variable ("present/absent") in all pathology reports, we attempted to retrospectively determine the grade by re-evaluating archival histological slides. However, for some earlier cases from the 2009–2013 period, original histological materials were no longer available in the archive, and in other cases, the available slides did not contain sufficient detail for reliable microscopic reassessment. Therefore, grading could be applied only to the 38 patients whose materials were fully accessible and suitable for evaluation. This represents another inherent limitation of the retrospective nature of this study.

Moreover, the statistical power of this study—particularly for the overall survival analysis—was inherently limited by the relatively small number of survival events and recurrence cases. The low event rate reduces the ability to detect modest but potentially clinically meaningful differences between groups. Therefore, the absence of a statistically significant survival difference should be interpreted cautiously, as it may reflect insufficient power rather than the true lack of an association.

Recent evidence has further highlighted the importance of integrating modern diagnostic and treatment approaches when interpreting nodal prognostic factors in breast cancer. Studies have shown that advances in breast imaging, molecular subtyping, and neoadjuvant therapy have substantially improved survival outcomes, particularly in patients with dense breast tissue or locally advanced disease [Clinics (Sao Paulo) 2025;80:100743; Clinics (Sao Paulo) 2024;79:100510; Front Oncol 2023;13:1293288]. These developments emphasize the need to contextualize nodal findings such as capsule invasion within a broader, multidisciplinary diagnostic and therapeutic framework.

Prior studies also linked extracapsular invasion in metastatic nodes to worse outcomes in various cancers^21,22^, including breast cancer^2^.

Capsular invasion should be routinely reported in breast cancer pathology, as it may influence recurrence. Further studies are needed to assess the prognostic significance of its extent and to establish a reliable cut-off value.

In our study, capsular invasion was significantly associated with an increased recurrence rate; however, this finding did not translate into a difference in overall survival. Several factors may explain this discrepancy. First, modern systemic therapies and radiotherapy regimens have substantially improved post-recurrence outcomes, potentially mitigating the survival impact of early locoregional failure. Second, many patients who experienced recurrence received effective salvage treatments, which may have equalized survival probabilities between groups. Third, the relatively limited number of recurrence events and the presence of advanced-stage disease in part of the cohort could have reduced the statistical power to detect survival differences. Additionally, the long study period (2009–2018) encompasses evolving treatment standards, which may have influenced long-term outcomes heterogeneously.

Notably, patients with capsular invasion had a higher mean number of malignant lymph nodes excised (7.43 vs. 3.92; p=0.020), which may have influenced survival outcomes. Additionally, grading of capsule invasion showed a significant correlation with recurrence, suggesting that not just the presence but also the severity of invasion may have prognostic relevance.

## Data Availability

The datasets generated and/or analyzed during the current study are available from the corresponding author upon reasonable request.

## References

[1] Ma X, Yang X, Yang W, Shui R (2021). Prognostic value of extranodal extension in axillary lymph node-positive breast cancer. Sci Rep.

[2] Freitas GB, Mota BS, Maesaka JY, Pinheiro CC, Lima LGCA, Soares JM (2023). Measurement of extracapsular extension in sentinel lymph node as a possible predictor of residual axillary disease in breast cancer. Clinics (Sao Paulo).

[3] Gabani P, Merfeld E, Srivastava AJ, Weiner AA, Ochoa LL, Mullen D (2019). Predictors of locoregional recurrence after failure to achieve pathologic complete response to neoadjuvant chemotherapy in triple-negative breast cancer. J Natl Compr Canc Netw.

[4] Almangush A, Mäkitie AA, Triantafyllou A, Bree R, Strojan P, Rinaldo A (2020). Staging and grading of oral squamous cell carcinoma: an update. Oral Oncol.

[5] Chen H, Meng X, Hao X, Li Q, Tian L, Qiu Y (2022). Correlation analysis of pathological features and axillary lymph node metastasis in patients with invasive breast cancer. J Immunol Res.

[6] Zhu M, Zheng W, Xiang Y, Gu J, Wang K, Shang J (2020). The relationship between central lymph node metastasis and the distance from tumor to thyroid capsule in papillary thyroid microcarcinoma without capsule invasion. Gland Surg.

[7] Kanyılmaz G, Fındık S, Yavuz BB, Aktan M (2018). The significance of extent of extracapsular extension in patients with T1-2 and N1 breast cancer. Eur J Breast Health.

[8] Fisher ER, Gregorio RM, Redmond C, Kim WS, Fisher B (1976). Pathologic findings from the national surgical adjuvant breast project. (Protocol no. 4). III. The significance of extranodal extension of axillary metastases. Am J Clin Pathol.

[9] Kustić D (2024). Size of extranodal extension in the sentinel lymph node as a predictor of prognosis in early-stage breast cancer. Clin Breast Cancer.

[10] Yang X, Ma X, Yang W, Shui R (2020). Clinical significance of extranodal extension in sentinel lymph node positive breast cancer. Sci Rep.

[11] Klarica Gembić T, Grebić D, Gulić T, Golemac M, Avirović M (2024). Predictive and prognostic values of glycoprotein 96, androgen receptors, and extranodal extension in sentinel lymph node-positive breast cancer: an immunohistochemical retrospective study. J Clin Med.

[12] Ahmad Z, Khurshid A, Qureshi A, Idress R, Asghar N, Kayani N (2009). Breast carcinoma grading, estimation of tumor size, axillary lymph node status, staging, and nottingham prognostic index scoring on mastectomy specimens. Indian J Pathol Microbiol.

[13] Nottegar A, Veronese N, Senthil M, Roumen RM, Stubbs B, Choi AH (2016). Extra-nodal extension of sentinel lymph node metastasis is a marker of poor prognosis in breast cancer patients: a systematic review and an exploratory meta-analysis. Eur J Surg Oncol.

[14] Katz A, Strom EA, Buchholz TA, Thames HD, Smith CD, Jhingran A (2000). Locoregional recurrence patterns after mastectomy and doxorubicin-based chemotherapy: implications for postoperative irradiation. J Clin Oncol.

[15] Nowikiewicz T, Wnuk P, Małkowski B, Kurylcio A, Kowalewski J, Zegarski W (2017). Application of artificial neural networks for predicting presence of non-sentinel lymph node metastases in breast cancer patients with positive sentinel lymph node biopsies. Arch Med Sci.

[16] Leonard C, Corkill M, Tompkin J, Zhen B, Waitz D, Norton L (1995). Are axillary recurrence and overall survival affected by axillary extranodal tumor extension in breast cancer? Implications for radiation therapy. J Clin Oncol.

[17] Gooch J, King TA, Eaton A, Dengel L, Stempel M, Corben AD (2014). The extent of extracapsular extension may influence the need for axillary lymph node dissection in patients with T1-T2 breast cancer. Ann Surg Oncol.

[18] Schwentner L, Dayan D, Wöckel A, Janni W, Kreienberg R, Blettner M (2018). Is extracapsular nodal extension in sentinel nodes a predictor for nonsentinel metastasis and is there an impact on survival parameters?-A retrospective single center cohort study with 324 patients. Breast J.

[19] Dobi E, Bazan F, Dufresne A, Demarchi M, Villanueva C, Chaigneau L (2013). Is extracapsular tumour spread a prognostic factor in patients with early breast cancer?. Int J Clin Oncol.

[20] Gruber G, Cole BF, Castiglione-Gertsch M, Holmberg SB, Lindtner J, Golouh R (2008). Extracapsular tumor spread and the risk of local, axillary and supraclavicular recurrence in node-positive, premenopausal patients with breast cancer. Ann Oncol.

[21] Chen D, Mao Y, Zheng Y, Wen J, Song P, Xue Y (2021). Extracapsular lymph node involvement is a robust survival predictor in esophageal cancer patients: a pooled analysis. Eur J Surg Oncol.

[22] Hartel P, Davey D (2022). Extracapsular lymph node tumour extension is a potential biomarker for immune-modulating therapy in colon cancer. Acta Sci Gastrointestinal Disord.

